# Comparison of dose-dense vs. 3-weekly paclitaxel and carboplatin in the first-line treatment of ovarian cancer in a propensity score-matched cohort

**DOI:** 10.1186/s12885-021-08270-0

**Published:** 2021-05-08

**Authors:** Rafaela Pirolli, Viviane Teixeira Loiola de Alencar, Felipe Leonardo Estati, Adriana Regina Gonçalves Ribeiro, Daniella Yumi Tsuji Honda, Mariana de Oliveira, Joao Paulo da Silveira Nogueira Lima, Elizabeth Santana dos Santos, Andrea Paiva Gadelha Guimarães, Glauco Baiocchi, Alexandre André Balieiro Anastácio da Costa

**Affiliations:** 1 Department of Medical Oncology, A.C. Camargo Cancer Center, 211 Professor Antonio Prudente Street, Liberdade, Sao Paulo, SP 01509-900 Brazil; 2 Department of Gynecologic Oncology, A.C. Camargo Cancer Center, 211 Professor Antonio Prudente Street, Liberdade, Sao Paulo, SP 01509-900 Brazil

**Keywords:** Ovarian cancer, Dose-dense, Chemotherapy, Weekly paclitaxel, Carboplatin

## Abstract

**Background:**

Benefit of carboplatin and dose-dense weekly paclitaxel (ddCT) in first line treatment of ovarian cancer patients has been different in Western and Asian studies. In the present study we compare progression-free survival (PFS) of ddCT to three-weekly carboplatin and paclitaxel (CT) in first-line treatment of ovarian carcinoma in a single institution in a Western population.

**Materials and methods:**

We conducted a retrospective review of medical records from patients with ovarian carcinoma treated in a tertiary cancer center from 2007 to 2018. All patients treated with ddCT or CT in the first-line setting were included. Patients who received first-line bevacizumab were not included. PFS and overall survival (OS) were compared in a propensity score-matched cohort to address selection bias. Patients were matched according to age, ECOG performance status, CA 125, FIGO stage, residual disease, and histological subtype, in a 1:2 ratio.

**Results:**

Five hundred eighty-eight patients were eligible for propensity score matching, the final cohort consisted of 69 patients treated with ddCT and 138 CT group. Baseline characteristics were well-balanced. After a median follow-up of 65.1 months, median PFS was 29.3 vs 20.0 months, favouring ddCT treatment (*p* = 0.035). In the multivariate cox regression ddCT showed a 18% lower risk of progression (HR 0.82, 95% CI 0.68–0.99, *p* = 0.04). Overall survival data is immature, but suggested better outcomes for ddCT (not reached versus 78.8 months; *p* = 0.07).

**Conclusion:**

Our retrospective study has shown superior PFS of ddCT over CT regimen in first-line treatment of ovarian carcinoma in a Western population not treated with bevacizumab.

## Implications for practice

The benefit of adjuvant dose-dense weekly paclitaxel and carboplatin over the conventional 3 weekly regimen in the present study of real word experience highlights the importance to further evaluate dose-dense regimen as an option to treatment of primary ovarian cancer patients in view of the limitations of previous phase 3 trials in western population.

## Introduction

Ovarian cancer is the seventh most common cancer in women, the eighth most common cause of cancer death around the world, and epithelial ovarian cancer (EOC) is the most lethal gynecologic cancer [1, 2]. Most ovarian cancer patients are diagnosed at FIGO stage III or IV and present a 5-year relative survival rate of 30% [3].

Primary debulking surgery (PDS) followed by intravenous administration of carboplatin at an area under the curve 5–6 (AUC) and paclitaxel 175 mg/m^2^ over 3 h every 3 weeks for six cycles is the standard of care in advanced EOC [4]. Currently, there are at least four treatment alternatives with better overall survival (OS) results in phase III studies, which include: carboplatin and dose-dense paclitaxel regimen [5, 6]; association of bevacizumab to carboplatin and paclitaxel, with better OS exclusively in women at high risk of progression (FIGO stage III disease with > 1 cm residual disease following PDS, and FIGO stage IV disease) [7, 8]; intraperitoneal chemotherapy [9–11]; and hyperthermic intraperitoneal chemotherapy (HIPEC) [12]. The latter two approaches are associated with a higher incidence of toxicity and hard to reproduce.

One large trial by the Japanese Gynecologic Oncology Group (JGOG3016) showed improvement in both progression-free survival (PFS) and OS with weekly administration of paclitaxel. In this study, 631 women with FIGO stage II–IV EOC were randomized to receive either carboplatin AUC 6 with paclitaxel 180 mg/m^2^ every 3 weeks, or carboplatin AUC 6 every 3 weeks with weekly paclitaxel 80 mg/m^2^ for up to nine cycles. Patients receiving dose-dense therapy compared with conventional treatment presented a significant improvement in PFS (28.2 vs. 17.5 months) and OS (100.5 vs. 62.2 months) [5, 6].

However, results from subsequent trials in the Western population, Gynecologic Oncology Group (GOG) 0262 and ICON8, did not replicate the benefit in OS to the previously reported data on survival [13, 14]. In GOG0262, 792 patients with FIGO stage II-IV EOC were randomly assigned to paclitaxel 175 mg/m^2^ and carboplatin AUC 6 every 3 weeks or weekly paclitaxel 80 mg/m^2^ with carboplatin AUC 6 every 3 weeks. Bevacizumab 15 mg/kg every 3 weeks was optional in both arms and was administered to 84% of patients. There was no difference in PFS among patients assigned to the dose-dense compared with the conventionally dosed chemotherapy group (14.7 vs 14.0 months). However, there was a difference in median PFS within the group of patients who did not receive bevacizumab in favor of the dose-dense arm (14.2 vs 10.3 months) [13]. The third phase 3 study ICON8 randomized 1566 patients with FIGO stage IC–IV EOC into 3 groups: carboplatin AUC 5 or 6 and paclitaxel 175 mg/m^2^ every 3 weeks (arm 1); carboplatin AUC 5 or 6 every 3 weeks and paclitaxel 80 mg/m^2^ weekly (arm 2); carboplatin AUC 2 and paclitaxel 80 mg/m^2^ weekly (arm 3). There was no statistically significant difference in PFS between the standard treatment group compared to the weekly treatment groups (17.7, 20.8 and 21.0 months respectively in arms 1,2 and 3) [14]. There are two other negative phase 3 trials evaluating weekly paclitaxel regimens, but in these studies the paclitaxel regimens and the study designs were quite different from the studies cited above [15, 16].

Despite the negative results, these studies have some differences and none of them had the same patient characteristics of the Japanese trial. Moreover, pharmacogenetics differences in Japanese versus Western populations could partially explain conflicting results.

In this study, we aimed to evaluate the effect of the dose-dense versus conventional regimen with carboplatin and paclitaxel, without bevacizumab, in a Brazilian population.

## Material and methods

### Patients

This retrospective cohort study comprised all consecutive patients with epithelial ovarian, tubal or peritoneal cancer who were treated at A.C. Camargo Cancer Center from January 2007 to December 2018, and who were treated with carboplatin plus weekly (dose-dense regimen) or 3-weekly paclitaxel (conventional regimen), irrespective of the date of initial diagnosis. In the present study we followed the same methodology for clinical data collection and statistical procedures as previously described in another paper of our group [17]. To address selection bias, we performed an analysis in a propensity score-matched cohort including all patients who were treated with dose-dense regimen and matching 1:2 to patients who were treated with a conventional regimen.

The study was conducted in accordance with the Declaration of Helsinki ethical guidelines and was approved by the Research Ethics Committee of A.C. Camargo Cancer Center (REC registry n° 2389/17). Informed consent was waived by the the Research Ethics Committee of A.C. Camargo Cancer Center (REC registry n° 2389/17) owing to the retrospective analysis of the study.

### Clinical data

In the present study we followed the same methodology for clinical data collection as previously described in another paper of our group [17]. Briefly, clinical findings were retrieved from the medical records. Baseline characteristics included age at diagnosis, date of initial diagnosis, tumor histological subtype, International Federation of Gynecology and Obstetrics (FIGO) stage, ECOG performance status, serum CA 125, moment of debulking surgery, residual disease after debulking surgery and *BRCA1/2* germline status.

Recurrence was defined per the GCIG (Gynecological Cancer Intergroup) criteria for CA125 progression or per RECIST (Response Evaluation Criteria in Solid Tumors) for image studies obtained from the medical records. The date of the earliest event was considered for progression. PFS was defined as the interval between the date of the diagnosis and the date of first recurrence or death due to any cause. OS was defined as the interval between the date of the diagnosis and death due to any cause.

### Treatment

Both study groups received carboplatin at a dose calculated to produce an AUC of 5 or 6 mg/mL per min on day 1 of a 21-day cycle. The standard group received paclitaxel given as a 3-h intravenous infusion at a dose of 175 mg/m^2^ on day 1 and in the dose-dense group, paclitaxel was administered as a 1-h intravenous infusion at a dose of 80 mg/m^2^ on days 1, 8, and 15 every 3 weeks. Patients needed to have an absolute neutrophil count of 1.0 × 10^9^ cells per L or more and a platelet count of 75 × 10^9^ per L or more to receive subsequent cycles of therapy in both groups. Patients in the dose-dense regimen group also had to have an absolute neutrophil count of 0.5 × 10^9^ cells per L or more and a platelet count of 50 × 10^9^ per L or more before they received paclitaxel on days 8 and 15.

The decision with regard primary or interval debulking surgery was made per surgeon with expertise in gynecologic oncology at our institution. There were no patients treated with PARP inhibitors or bevacizumab.

### Statistical analysis

To overcome possible selection bias between dose-dense and conventional chemotherapy regimen patients, we performed a propensity score matching (PSM) with a 1:2 ratio. The PSM overcomes the different distribution of covariates among individuals allocated to specific interventions in the study. All patients treated with dose-dense regimen were identified and each one of them was matched to 2 patients treated with the conventional regimen with the closest propensity score using greedy matching and the nearest neighbor matching approaches. The propensity score model was generated using all potential covariates that could affect the group allocation aiming to draw more reliable results. Patients were matched according to age younger or older than 65 years old, ECOG performance status of 0 or ≥ 1, CA 125 less or more than 200, FIGO stage I-II or III-IV, primary or interval surgery, residual disease and histological subtype.

In the present study we followed the same methodology for other statistical procedures as previously described in another paper of our group [17]. Briefly, frequencies, medians and interquartile range (IQR) were used to describe patients’ characteristics. The association between patients’ characteristics was tested by Qui-square Test. PFS and OS curves were plotted by Kaplan-Meier method and we used log-rank test to test the association between clinical characteristics and survival. Hazard ratios for PFS were calculated by Cox regression analysis, variables with *p* <  0.2 in the univariate analysis were included in the multivariate analysis.

The statistical analysis was performed using SPSS v. 24.0 (SPSS, Chicago, IL, US) and R program, adopting a two-tailed *p* value < 0.05 as statistically significant.

## Results

**Fig. 1:**
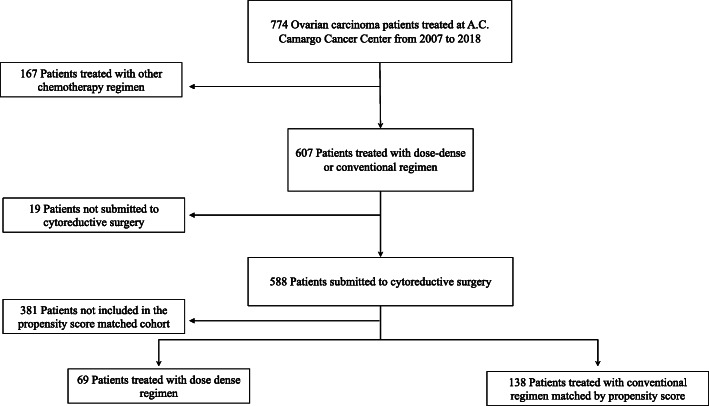
Flowchart of patients’ inclusion in the study

Seven hundred and seventy-four patients with ovarian cancer were treated in our institution from 2007 to 2018. After excluding patients who were not submitted to cytoreductive surgery and who were treated with other chemotherapy regimens, 69 patients were treated with dose-dense carboplatin and paclitaxel and 519 patients were treated with 3-weekly carboplatin and paclitaxel, making a total of 588 patients eligible for propensity score matching at a 1:2 ratio. The final matched cohort consisted of all 69 patients in the dose-dense group and 138 patients in the conventional chemotherapy group (Fig. 1).

For the role cohort, median age was 54.3 years old, 60.8% of patients presented ECOG performance status of zero, 83.1% of tumors were high-grade serous carcinoma, FIGO stage was I in 4.8%, II in 2.4%, III in 82.6% and IV in 10.1% of patients. Sixty-seven percent of patients were treated with primary debulking surgery and complete cytoreduction was achieved in 88% of patients. Twenty patients (9.8%) presented limiting toxicity leading to dose reductions or treatment interruption, 14 (20.6%) in the dose-dense group, and 6 (4.4%) in the 3-weekly group (*p* <  0.001). Eight patients (3.9%) discontinued adjuvant chemotherapy before 6 cycles due to toxicity, 3 (4.3%) in the dose-dense group and 5 (3.6%) in the 3-weekly group (*p* = 0.870).

Baseline characteristics of patients in both groups are shown in Table 1. The two groups were well balanced and there were no significant differences between them. Compared to patients who were treated with conventional regimen, patients treated with dose-dense protocol had a somewhat higher proportion of high-grade serous carcinoma (88.4% vs. 80.4%), better performance status (ECOG 0 65.2% vs. 57.2%) and lower levels of CA125 (CA125 < 200 in 42% vs. 37.7%) but none of these differences were statistically significant. Data on *BRCA1/2* mutational status was not available for a large number of patients, however, among the tested patients, the difference in the frequency of mutations between groups was not statistically significant.

**Table 1:** Baseline characteristics

Characteristic		Freq. (%)	
	Dose-dense regimen (***n*** = 69)	Conventional regimen (***n*** = 138)	***p***^**a**^
Age			
Median (P25–75)	52.6 (45.5–59.1)	54.8 (47.3–61.3)	0.290
≤ 65	64 (92.8)	128 (92.8)	1.000
> 65	5 (7.2)	10 (7.2)	
FIGO stage			
I -II	5 (7.2)	10 (7.2)	1.000
III-IV	64 (92.8)	128 (92.8)	
Histology			
High-grade serous carcinoma	61 (88.4)	111 (80.4)	0.149
Others	8 (11.6)	27 (19.6)	
ECOG performance status			
0	45 (65.2)	79 (57.2)	0.134
≥ 1	21 (30.4)	59 (42.8)	
Missing data	3 (4.3)	0 (0)	
CA125			
Median (P25–75)	344 (72.8–1427)	392.8 (97–1470.2)	0.614
≤ 200	29 (42)	52 (37.7)	0.442
> 200	38 (55.1)	86 (62.3)	
Missing data	2 (2.9)	0 (0)	
Type of cytoreductive surgery			
Primary	48 (69.6)	92 (66.7)	0.674
Interval	21 (30.4)	46 (33.3)	
Complete cytoreduction			
Yes	61 (88.4)	120 (87)	0.570
No	7 (10.1)	18 (13)	
Missing data	1 (1.4)	0 (0)	
*BRCA1/2* mutation			
Yes	14 (20.3)	11 (8)	0.793
No	44 (63.9)	39 (28.2)	
Missing data	11 (15.9)	88 (63.8)	
Family history of breast or ovarian cancer			
Yes	21 (30.4)	46 (33.4)	0.778
No	43 (62.4)	86 (62.3)	
Missing data	5 (7.2)	6 (4.3)	

^a^Used to compare valid data only. All *p* values calculated by chi square test

For the 67 patients treated with neoadjuvant chemotherapy, data on response was available for 62 patients and the overall response rate (ORR) was 90.3%. The ORR was 90.7% for the dose-dense group and 89.5% for the conventional chemotherapy group (*p* = 1.000).

### Progression-free survival

**Fig. 2:**
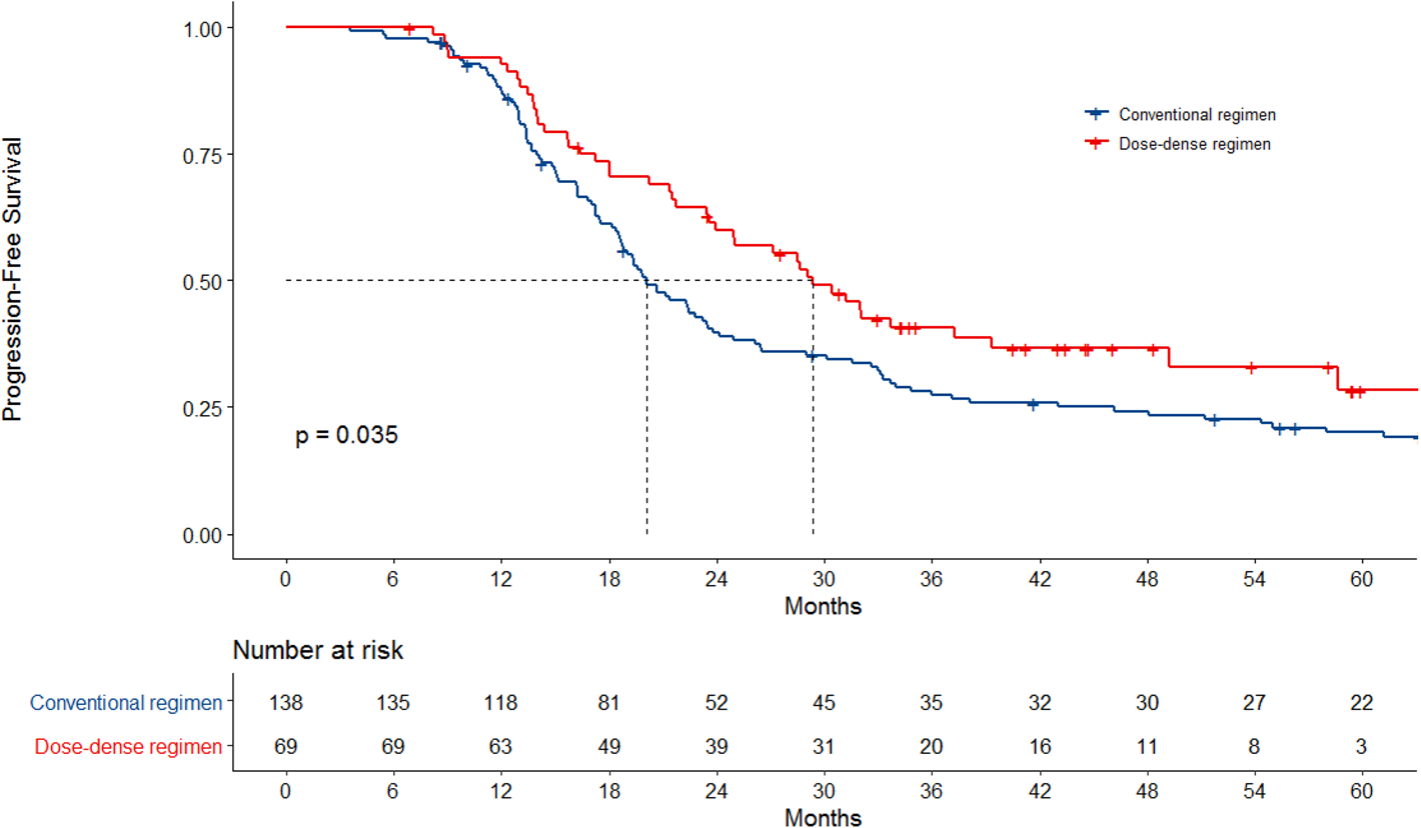
Progression-free survival according to chemotherapy regimen

After a median follow-up of 65.1 months, 154 progression events happened, and the median PFS was 23.2 months. Median PFS was longer for patients who were treated with dose-dense regimen compared to patients who were treated with conventional regimen, with 29.3 vs 20.0 months (*p* = 0.035) (Fig. 2). PFS at 3 years was 40.8% vs 27.4%.

In univariate analysis PFS was significantly longer in the dose-dense group (HR = 0.82; 95% CI 0.69–0.98; *p* = 0.036). Other variables associated with longer PFS were FIGO stage I-II, CA125 lower than 200, primary cytoreductive surgery and no residual disease after surgery. In multivariate cox regression dose-dense regimen showed a 18% lower risk of progression (HR 0.82, 95% CI 0.68–0.99, *p* = 0.040). Interval debulking surgery (HR 1.76, 95% CI 1.18–2.63, *p* = 0.005) and residual disease after surgery (HR 2.0, 95% CI 1.22–3.24, *p* = 0.006) also remained as independent prognostic factors in the final model (Table 2).

**Table 2:** Univariate and multivariate analysis for progression-free survival

Characteristic	Univariate		Multivariate	
	HR (95% CI)	***p*** value	HR (95% CI)	***p*** value
Age at diagnosis				
≤ 65	1	0.412		
> 65	0.76 (0.40–1.45)			
Stage				
I -II	1	0.042	1	0.222
III-IV	2.10 (1.03–4.28)		1.59 (0.75–3.36)	
Histology				
High-grade serous carcinoma	1	0.125	1	0.757
Others	0.70 (0.44–1.10)		0.92 (0.57–1.51)	
ECOG performance status				
0	1	0.089	1	0.790
≥ 1	1.32 (0.96–1.83)		0.95 (0.66–1.37)	
CA 125 at diagnosis				
≤ 200	1	0.046	1	0.581
> 200	1.40 (1.01–1.94)		1.10 (0.78–1.56)	
Type of cytoreductive surgery				
Primary	1	< 0.001	1	0.005
Interval	2.02 (1.44–2.82)		1.76 (1.18–2.62)	
Complete cytoreductive surgery				
Yes	1	< 0.001	1	0.006
No	2.64 (1.65–4.23)		1.99 (1.22–3.24)	
Chemotherapy regimen				
Conventional	1	0.036	1	0.040
Dose-dense	0.83 (0.69–0.99)		0.82 (0.68–0.99)	
*BRCA1/2* mutation				
No	1	0.766		
Yes	1.08 (0.66–1.82)			
Family history of breast or ovarian cancer				
No	1	0.916		
Yes	0.98 (0.70–1.38)			

### Overall survival

**Fig. 3:**
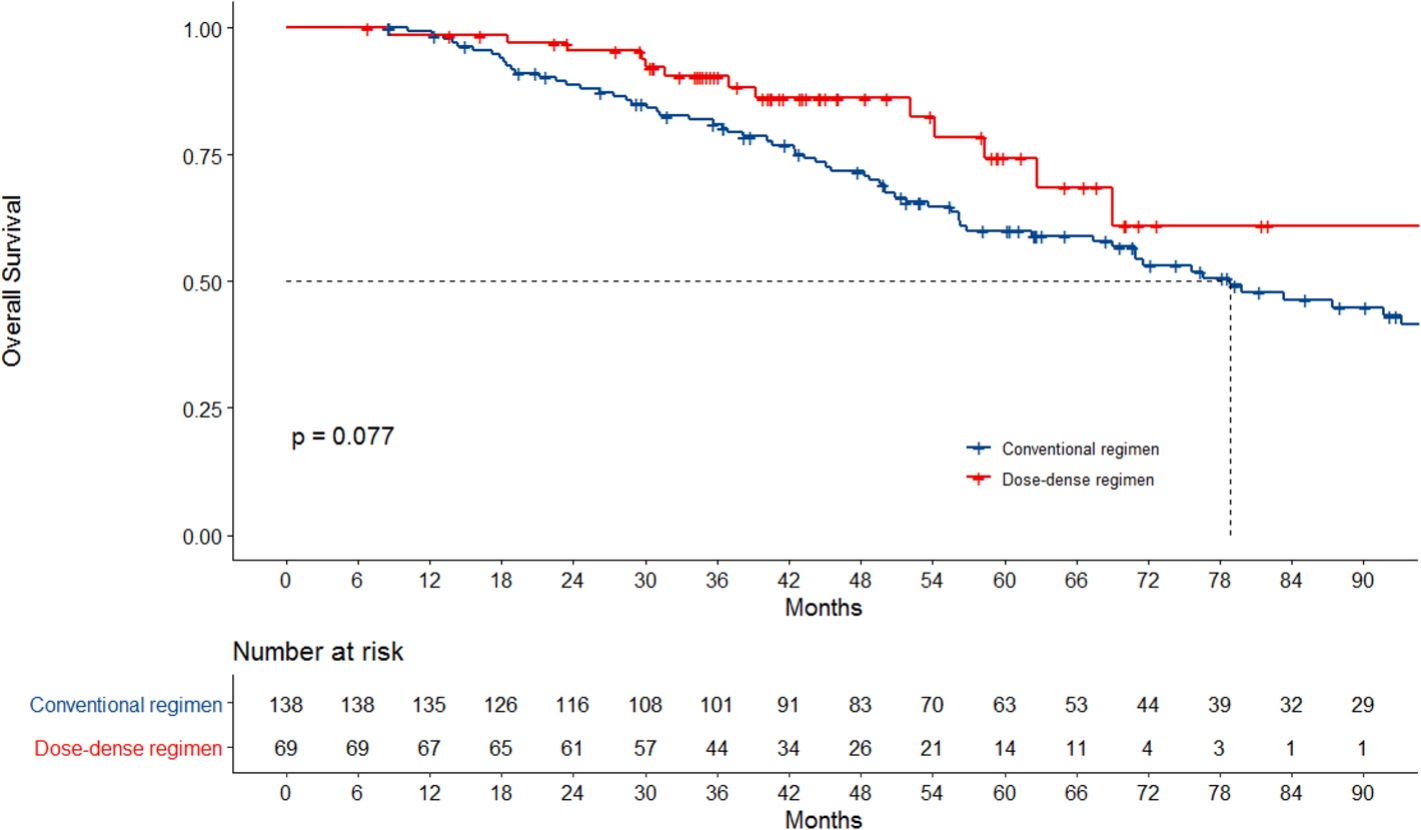
Overall survival according to chemotherapy regimen

Eighty-one deaths happened and median OS for all patients was 83.3 months. There was no statistically significant difference in median OS between patients who were treated with dose-dense regimen compared to patients who were treated with conventional regimen, with an OS not reached vs 78.8 months, (*p* = 0.07), although longer follow-up is needed (Fig. 3). OS at 3 and 5 years were 90.4% vs 81.0, and 74.4% vs 60.1% respectively in favor of dose-dense regimen.

### Subgroup analysis

**Fig. 4:**
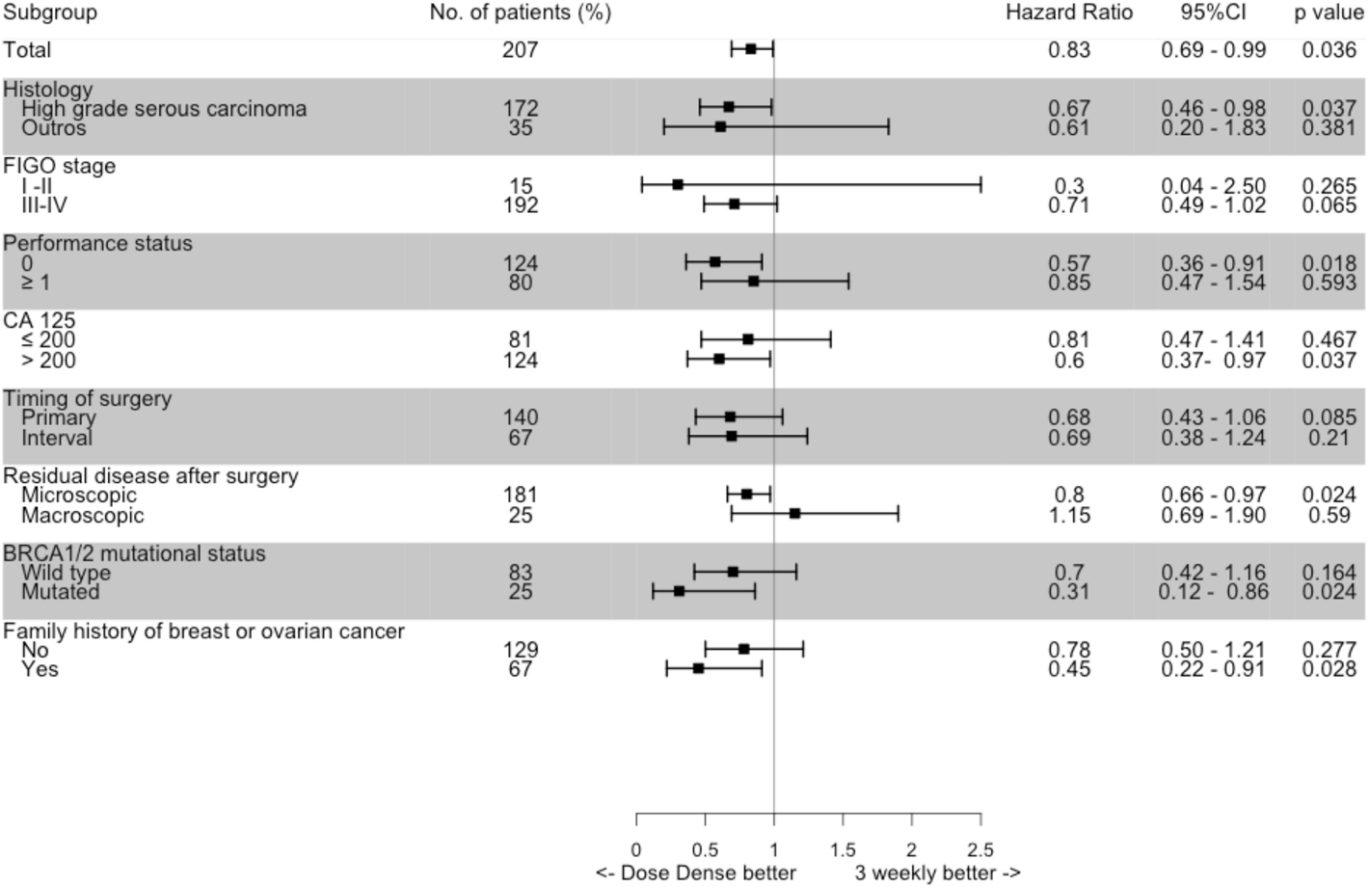
Hazard ratios for progression-free survival by baseline characteristics

In a subgroup analysis for PFS the benefit of dose-dense regimen was homogeneous among most characteristics. Patients who presented *BRCA1/2* mutations presented a greater benefit from dose-dense than wild-type patients, as well as patients with positive family history for breast or ovarian cancer presented a greater benefit from dose-dense than patients who did not have family history of breast or ovarian cancer (Fig. 4).

## Discussion

Ideal first-line treatment of advanced ovarian cancer remains disputable, 3-weekly carboplatin and paclitaxel is considered the standard of care and addition of bevacizumab, use of intraperitoneal chemotherapy or dose-dense paclitaxel have emerged as alternative treatment strategies [4]. Despite the OS survival benefit of dose-dense schedule showed by JGOG3016, the negative results from GOG0262 and ICON8 put into question the benefit of dose-dense paclitaxel in Western populations [5, 6, 13, 14]. In this retrospective study, we showed a longer PFS for patients who were treated with dose-dense regimen compared to patients who received conventional regimen, with PFS of 29.3 vs 20.0 months (*p* = 0.035), and 18% less risk of progression on multivariable analysis. Although OS data is still immature, there was a non-statistically significant benefit in favor of the dose-dense group (NR versus 78.8 months; *p* = 0.07).

PFS results in the present study reproduce the results from JGOG3016 in both arms that showed median PFS of 28.2 months vs 17.5 months in favor of dose-dense regimen [5, 6]. The median PFS of 20.0 months in the 3-weekly group in our study is also similar to the 3-weekly arm from ICON8 trial [14]. Median PFS in GOG0262 was shorter in both arms (14.7 months vs 14.0 months) probably related to the higher frequency of FIGO stage IV disease, which was present in 30% of patients, and the higher frequency of macroscopic residual disease after surgery, which was present in 63% of patients [13].

Patients in our study have not been treated with bevacizumab in first-line and only 30% were treated with neoadjuvant chemotherapy. This makes our patients’ characteristics and treatment schedules more similar to JGOG3016 than GOG0262 and ICON8 trials that differ from the Japanese study by the use of bevacizumab in both arms of GOG0262 and the high proportion of patients treated with neoadjuvant chemotherapy in ICON8 [5, 6, 13, 14].

A dose-dense regimen of paclitaxel involving greater frequency of drug delivery may improve its antineoplastic effect by angiogenesis inhibition and enhanced intra-tumoral drug perfusion [18, 19]. This could explain why no difference in PFS was observed in GOG0262 study where only 16% of the total did not receive bevacizumab. In the subgroup of patients who did not receive bevacizumab, weekly paclitaxel was associated with longer PFS than was paclitaxel administered every 3 weeks, with 38% less risk of progression or death [13].

Weekly paclitaxel may provide dividing tumor cells with more sustained exposure to paclitaxel compared with standard doses every 3 weeks. Hence, dose-dense approach could minimize tumor regrowth between cycles and limit the emergence of chemotherapy resistant cells [19]. In ICON8 trial 50% of patients underwent interval debulking surgery (IDS) after neoadjuvant chemotherapy. The pause of chemotherapy to proceed IDS may hamper this benefit of dose-dense schedule. Furthermore, patients selected to IDS have bulkier disease and worse prognosis and a recent study revealed that interruption of chemotherapy before and after IDS for more than 10 weeks was associated with worse prognosis [20]. Indeed, in the subgroup of patients treated with primary surgery, the survival curves do split but the study was underpowered for this subgroup analysis. In addition, there is no data on residual disease status after primary surgery, and an unbalanced distribution of residual disease status between groups may have impacted the results [14].

Two other prospective studies showed no benefit with weekly paclitaxel [15, 16]. MITO-7 trial evaluated weekly paclitaxel at a dose of 60 mg/m^2^ and carboplatin in patients with FIGO stage IC–IV EOC and did not show a survival benefit [15]. Another trial evaluated patients with FIGO stage IIB–IV EOC and randomized them to standard 6 cycles of three weekly carboplatin and paclitaxel or a schedule consisted of paclitaxel 90 mg/m^2^ and carboplatin AUC 4 or cisplatin 70 mg/m^2^ weekly on days 1, 8, 15, 29, 36, and 43; then there was a second randomization for three weekly carboplatin and paclitaxel for 3 versus 6 cycles. There was no survival benefit for the weekly regimen neither for extended chemotherapy [16]. Both trials used weekly paclitaxel schedules that are different from the dose-dense chemotherapy as defined in the present study and in the Japanese trial, hampering any comparison to the other studies of dose-dense chemotherapy.

Molecular characteristics such as *BRCA1* or *BRCA2* mutational status, homologous recombination deficiency (HRD) or molecular subtypes based on gene expression signatures may impact prognosis [21, 22]. It is possible that differences in the proportion of *BRCA1/2* mutated patients, HRD and different molecular subtypes may have contributed to different results of PFS and OS observed in the trials [23]. In our study *BRCA1/*2 germline mutational status was available for only 52.2% of patients and it was not related to prognosis. Interestingly patients with *BRCA1/*2 mutations seemed to benefit more from the dose-dense regimen. While preclinical data would suggest *BRCA1/2* mutated tumors are more resistant to paclitaxel [24] and data on antiangiogenic trials suggest that *BRCA1/*2 mutations and HRD did not predict antiangiogenic benefit [25, 26], at least one small retrospective study showed *BRCA1/2* mutations are sensitive to paclitaxel monotherapy [27]. These findings should be interpreted with caution due to the small number of patients in the subgroups, but there is scarce data in the literature addressing the association of *BRCA1/*2 mutations and the benefit of dose-dense regimen. Recently, an exploratory analysis of the VELIA study showed longer PFS with a dose-dense schedule in the overall cohort and in the subgroup evaluation the benefit was restricted to patients without *BRCA1/2* mutation and non HRD [28]. Post-hoc analysis in the other dose-dense studies should help to clarify this point.

It has been suggested that the pharmacogenomics differences between Japanese and Western populations are a possible cause for differences in outcomes between JGOG3016 and other studies. Efficacy and toxicity of paclitaxel are determined by drug exposure, and genetic variations may contribute to the variability of paclitaxel pharmacokinetics and pharmacodynamics. There are ethnic differences in polymorphisms of genes involved in the metabolism of paclitaxel, especially in CYP2C8 and CYP3A4/5 enzymes [23]. There are few data about distribution of pharmacogenetics polymorphisms among Brazilians, besides the fact that the Brazilian population is highly heterogeneous and admixed, which may cause high heterogeneity of drug responses [29]. A small study showed that the frequency of CYP2C8*2, for example, is similar to other registries, being more common in Brazilian black population (13% of the sample) and rare for white and Asian Brazilians (1 and 0%, respectively) [30]. The exact role and clinical impact of the pharmacogenetic ethnic variations have yet to be clarified, and an improved understanding about this topic may eventually help to explain the conflicting results between the studies.

Limiting treatment toxicities leading to dose reductions or treatment discontinuation was less frequent than expected in our study, about 10%, compared to the more than 30% rate of treatment discontinuation before 6 cycles in JGOG3016 and ICON8. These could be in part due to a younger patient population in our study with a median age of 54 years old compared to 63 years old in ICON8 and 57 years old in JGOG3016, but also could be due to a limitation of the retrospective nature of the study. Despite that, more patients in the weekly paclitaxel group presented limiting toxicity compared to the 3-weekly regimen in our study, as in the phase 3 trials [5, 6, 14].

The limitations of our study include its design as a retrospective study, the possibility of selection bias and the high frequency of missing data on *BRCA1/2* status. We addressed these limitations using propensity score matching according to age, ECOG performance status, CA 125, FIGO stage, primary or interval surgery, residual disease, and histological subtype. This led to two groups with balanced baseline characteristics. Moreover, the benefit of dose-dense paclitaxel persisted in cox multivariate analysis. Despite that, as we conducted an observational, non-randomized study we cannot rule out the risk that the patients in our two study groups were not comparable with regard to other variables that we did not account for.

## Conclusion

Our study is the first study in a Brazilian population to show the benefit of the dose-dense regimen when compared to standard 3-weekly paclitaxel, similar to JGOG3016 trial. While drug development in ovarian cancer is heading towards incorporation of new targeted drugs and immunotherapy in first-line treatment, we believe the data of ICON8 and GOG0262 do not offer enough evidence to rule out dose-dense chemotherapy as an adjuvant treatment option for non-Asian patients, and it should be considered as a possible backbone for new treatment regimens.

## Data Availability

The datasets used and/or analyzed during the current study are available from the corresponding author on reasonable request.
